# Hypnoptism, Mesmorism, or Syggnosticism

**Published:** 1881-04

**Authors:** 


### EDITORIAL DEPARTMENT.

"If thou hast truth to utter, speak;
And leave the rest to God."

Hypnotism, Mesmerism, or Syggnosticism—Has been revived recently in New York, and is attracting considerable attention, both in scientific and social circles; mainly from the fact that prominent gentlemen in the profession have taken it in charge, and brought it before the public again, through the influence of their high positions. But interesting, and almost startling, as some of the manifestations produced by Prof. Hammond, and Dr. Beard are, they were well known to many physicians, who, a quarter of a century ago, investigated for themselves, phenomena, which itinerant Mesmerisers were accustomed to parade for money before such audiences as their skill or juggling was able to command. In the hands of such gentlemen as are now investigating and practicing its mysteries, we may reasonably look forward to the discovery or development of new and startling phenomena. In fact, they have not as yet exhibited, if they have reached them in their investigations, spectacles which are more than marvelous, and actually transcend credulity until witnessed again and again under the most convincing circumstances. Some of Dr. Hammond's subjects seem to have passed the boundaries of the knowable and known, and reached the mysterious confines where the mesmerised can be made to relate with accuracy what is transpiring miles away, in regard to some persons and things of which he had neither personal knowledge nor acquaintance before he passed into what was formerly called the clairvoyant state. We recall the cases of persons, of a quarter of a century ago, which seem so strange at this day, and to border so much on the supernatural, that we can hardly credit ourselves with having had any agency in bringing about or developing in them this most marvelous condition.

But we did not intend to give our experience in this mysterious profundity, in an editorial, further than to endorse the truthfulness of the phenomena, which the learned gentlemen we have named, are practicing in the interest of Science; and if any word of ours would likely urge them in still further explorations of the arcana which nature seems willing now to yield to those who press earnestly for them, we would say with all confidence in the final result, go on with the great work, the prospects are bright for a munificient reward.

